# Assessing Equine Behavioural Responses in Equine-Assisted Services: A Field Study Analysis

**DOI:** 10.3390/ani15050671

**Published:** 2025-02-25

**Authors:** E. Kathalijne Visser, Anna L. Jens, Lydia E. Nieuwe Weme, Ayella A. Spaapen, Kyra N. Maarleveld, Kitty H. Enzerink, Pieter N. Tromp, Sandra C. Haven-Pross

**Affiliations:** Department of Applied Research, Aeres University of Applied Sciences Dronten, De Drieslag 4, 8251 JZ Dronten, The Netherlands; a.jens@aeres.nl (A.L.J.); l.nieuweweme@aeres.nl (L.E.N.W.); a.spaapen@aeres.nl (A.A.S.); k.maarleveld@aeres.nl (K.N.M.); k.enzerink@aeres.nl (K.H.E.); pieter.tromp@gmail.com (P.N.T.); s.haven@aeres.nl (S.C.H.-P.)

**Keywords:** horse–human interaction, welfare, equine-assisted services, health and safety, affective states

## Abstract

Equine-Assisted Services (EAS) offer significant benefits for individuals with intellectual and/or physical disabilities. However, ensuring the welfare of the horses involved remains a key ethical concern. The objective of this study was to assess the affective states of horses involved in EAS while also establishing behavioural thresholds for indicators of positive or negative affective states. A total of 98 horses were observed over a two-month period, accumulating data about horse behaviour and session characteristics from 830 EAS sessions. Horses participating in coaching programs averaged four sessions in two months; those involved in therapeutic riding averaged 31 sessions. Equine behaviour and field experts defined behavioural thresholds to assess welfare standards. Negative state behaviours were predominantly aligned within excellent or neutral thresholds, while positive state behaviours were observed from unacceptable to excellent. Principal Component Analysis (PCA) of the gathered behavioural data identified six affective states in coaching sessions and four in therapeutic riding, explaining 63% and 73% of the total variation, respectively. Among these states, “comfortable” was the most frequently observed. Multiple linear regression showed several significant variables, highlighting the importance of health, suitable horse selection, and proper management in promoting positive affective states in EAS.

## Simple Summary

Equine-Assisted Services (EAS) offer many benefits for people with intellectual or physical disabilities. As these services grow in popularity, ensuring the welfare of the horses involved is crucial. This study explored the horses’ mood state during equine-assisted coaching and therapeutic riding sessions, focusing on factors like session length, the horse’s personality, and care practices. The study included 98 horses which were followed over a two-month period where all EAS sessions were monitored, resulting in 830 EAS sessions. Horses in equine-assisted coaching typically participated in four sessions over two months, while those in therapeutic riding programmes attended 31 sessions on average. Equine behaviour and field experts set standards for evaluating horse behaviours. Behaviours suggesting discomfort were limited and mostly within acceptable levels, while the exhibited behaviours linked to positive affective states varied widely between horses and were seen in too few sessions. Furthermore, the study identified different affective states in horses, with “comfortable” being the most common state across both coaching and therapeutic riding. This study highlights the importance of maintaining horse health, selecting suitable horses, and implementing effective care practices to promote positive affective states in EAS.

## 1. Introduction

Equine-Assisted Services (EAS) encompass a range of approaches, all connected by the common element of involving horses. Equine-assisted coaching is a structured intervention aimed at enhancing personal development, where a licensed professional facilitates interactive growth in participants. In these sessions, horses are typically actively engaged in groundwork exercises, such as leading, obstacle navigation, or free interaction in an arena, allowing participants to observe and respond to the horse’s reactions. The horse may also be worked in a round pen or guided through specific tasks that encourage self-reflection and behavioural awareness. In some cases, participants interact with the horse through touch, movement, or guided activities designed to elicit emotional and cognitive insights [1]. Therapeutic riding, on the other hand, is specifically designed for individuals with disabilities, with certified instructors who customize horsemanship techniques to accommodate various needs [2].

Recent advancements in EAS have contributed to increasing attention and support from the scientific community, highlighting a growing body of empirical evidence demonstrating their effectiveness [3,4,5,6,7]. Studies have shown positive outcomes for participants across various age groups and disabilities, including children, adolescents, adults, and the elderly [8,9,10,11,12,13,14,15]. Additionally, systematic reviews have highlighted psychological benefits [4,7] and improvements in the physical function of human participants [16,17,18].

While EAS continue to gain popularity for therapeutic benefits to human participants, the welfare of the horses involved remains a critical area of focus. Horses, as sentient beings, are exposed to various physical and emotional stressors; they need to cope with human interactions [19]. This regulation is essential not only for the safety and efficacy of interventions but also for ethical considerations associated with their use. Research has identified a lack of comprehensive studies examining horses’ stress responses within EAS [20,21], urging calls for a multidimensional approach to assess horses’ reactions and prioritize their well-being in EAS. Complementary findings of a systematic review on equine time budgets [22] emphasize the importance of understanding horses’ natural behaviours and how deviations in their routine activities such as EAS can impact their welfare.

The well-being of horses in EAS is crucial for the safety of both humans and horses, and for the quality and efficacy of the interventions. Horses may experience stress from physical constraints and interactions with riders, particularly when riders have disabilities that lead to unpredictable, inconsistent, or excessive cues [23]. This underscores the importance of evidence-based practices, such as assessing indicators of stress and relaxation, to ensure horses are not negatively affected by their participation in EAS.

Environmental factors, including housing, diet, interactions with other horses, and horse–rider interactions, such as rider asymmetries, incorrect communication, or poor technique, also play a significant role in equine welfare [24,25]. In a systematic review, Hall and Kay [26] report that signs of negative affective states in horses include agonistic intra-specific social interactions, avoidance, and withdrawn and agitated behaviours, often resulting from inadequate social and physical environments. To address these possible additional challenges, it is vital to develop guidelines and standards that ensure horses are willing participants, their physical and emotional needs are met, and they are protected from excessive stress during EAS activities.

De Santis, Contalbrigo, Borgi, Cirulli, Luzi, Redaelli, Stefani, Toson, Odore, Vercelli, Valle, and Farina [20] emphasized the importance of continuing to use a multifaceted approach in future research on the welfare of equids in EAS. Similarly, Rankins et al. [27] conducted a comprehensive review on the use of equids in EAS and the ways in which welfare has been addressed to date. They argued that a multifaceted approach offers a deeper and more nuanced understanding of equine welfare and well-being. Such an approach is essential for tackling key challenges, including the discrepancies between behavioural and physiological indicators, the thorough reporting of stress responses and affective states, and the complexity of the EAS work environment.

Previous studies in EAS have evaluated horse welfare by examining isolated behaviours and focusing on indicators of poor welfare. However, as Boissy et al. [28] stress, this approach is problematic because the absence of poor welfare does not necessarily indicate the presence of good welfare. In line with the Five Domains Model framework [25], researchers and practitioners should also document behaviours that indicate positive affective states. A positive balance between positive and negative affective states is necessary to demonstrate that a horse can be argued to be truly in a state of good welfare. To describe affective states in animals, a two-dimensional framework has been suggested [29]. This approach characterizes animals’ affective state based on two key dimensions: valence (such as positive or negative, pleasant or unpleasant) and arousal/intensity level (such as contentment versus excitement). This framework confirms the need to explore affective states in EAS, where both positive and negative states impact horse welfare.

Identifying affective states in horses is essential for assessing their well-being, and this can be achieved through various methodologies, including data-driven approaches like Principal Component Analysis (PCA) and expert-defined behavioural thresholds, which help categorize and evaluate emotional states based on observed behaviours. PCA is widely used in equine behaviour research to identify patterns in temperament, emotional responses, and welfare-related behaviours. By reducing complex behavioural datasets into key components, PCA reveals hidden associations not evident through direct observation. In a large-scale study on UK leisure horses, PCA was used to categorize behaviour problems into meaningful dimensions, highlighting links between stable-related stress responses, pre-feeding aggression, and ridden behavioural issues [30]. Similarly, PCA has been employed to quantify temperament in young horses, identifying core personality traits that influence trainability and emotional reactivity [31]. These studies underscore the importance of PCA in understanding horse behaviour, providing valuable insights to enhance their involvement in equine-assisted services.

The current study aimed to assess the positive and negative affective states of horses during two different types of EAS—equine-assisted coaching and therapeutic riding lessons—emphasizing the connections between these states and various aspects such as the characteristics of the session itself, the individual horse, and management practices. Additionally, this study aimed to establish behavioural thresholds for behaviours indicative of positive or negative affective states. The overarching goal is to improve the chances of a good quality of life for horses participating in EAS.

## 2. Materials and Methods

This research study was conducted in collaboration with the Dutch Equine-Assisted Services (EAS) sector. It consisted of two components: a field study and an evaluation exercise aimed at defining behavioural thresholds relevant to EAS. In the field study, subjects from 37 distinct companies in the Netherlands participated, while the evaluation exercise involved subjects from 22 companies. Three companies contributed subjects to both the field study and the evaluation exercise.

This research was carried out in alignment with the Declaration of Helsinki and received approval from the Data Protection Officer at Aeres University of Applied Sciences, Dronten. Ethical review and approval for animals were waived for this study in compliance with the directive and current Dutch laws, as the experiments involved only behavioural observations and non-invasive interactions with the horses. The horses involved in this research were not classified as research animals (AER2022-02).

### 2.1. Field Study

Subjects were recruited through the project partners’ network and supplemented through advertisements in social media groups. All subjects, whether a therapeutic horse-riding instructor or a coach, received structured training in a classroom setting on utilizing an ethogram to identify and monitor horse behaviours observed during therapeutic horse-riding lessons or coaching sessions. This retrospective scoring methodology was selected to align with real-life scenarios where coaches or instructors can only assess behaviour after the session. This approach was also employed by the authors of [32], where judges were asked to evaluate behaviour following a dressage test.

#### 2.1.1. Ethogram

The ethogram was developed through the detailed analysis and scoring of 35 videotaped EAS sessions (therapeutic riding and coaching) collected in a previous study, utilizing the Observer software (Noldus Observer XT15) to document the range of behaviours observed and assess their frequency or duration. The study incorporated a total of 21 behaviours, carefully chosen to represent both behaviours potentially indicative of negative and positive affective states, and feasible to train and monitor in practice (see Table 1). Based on the videotapes, criteria for each specific behaviour were set to define the scoring categories: absent, observed occasionally, or observed frequently.

#### 2.1.2. Training

Equine-assisted coaches and therapeutic riding instructors were trained to document the frequency or duration of the 21 behaviours they observed post-session using three categories: absent, observed occasionally, and observed frequently. The training followed a two-stage process. First, coaches and therapeutic riding instructors were trained to identify behaviours as defined by the researchers using a method similar to that applied by Mullard, Berger, Ellis, and Dyson [38], where students determined the presence or absence of specific behaviours in video clips. Second, they learned to categorize these behaviours. They were shown video recordings of complete sessions and guided in retrospectively scoring behaviours within predefined categories (absent, observed occasionally, observed frequently). This approach ensured that all coaches and therapeutic riding instructors developed a consistent method for behaviour recognition and categorization before conducting field observations. The training concluded with instructions on using an app to document the observed behaviours after the sessions. This training was delivered in a classroom setting, either in-person or online. Upon covering the ethogram’s behaviours, coaches and riding instructors were required to take a test involving video clips of all listed behaviours. Passing the test required scoring at least 75% accuracy, which is similar to the expert training in the study of Johnson et al. [53] and student training in the study of Rankins et al. [54]. This training, including the test, took on average six hours per person. In total, six training sessions were held between September 2022 and April 2023.

#### 2.1.3. Collection of Data

Trained subjects were approved to access the app environment, where they could monitor and report after each EAS session that they delivered. This app was developed as a scoring method using Microsoft PowerApps (Version. 23081). Each subject monitored one or more horses continuously for two months, with every session of each horse being monitored and scored. All horses were monitored between October 2022 and July 2023.

In addition to the behaviour data of the horses during the session, other data were recorded: characteristics of the horse (33 questions), the session/client (17 questions), and the subject (6 questions).

Characteristics of the horse included breed, height at the withers, age, sex, coat colour, known medical problems, preventive health management such as vaccinations, deworming and farrier visits, housing management, feeding management, and equine personality profile. The questions related to housing and feeding management are derived from an equine welfare monitoring system designed for the Dutch horse sector [55]. The equine personality profile was obtained using the equine personality test (EPT) questionnaire developed by Ijichi et al. [56], which provided scores for agreeableness, extraversion, and neuroticism.

Characteristics of the session included date, duration, type of activity, familiarity with the horse, work earlier on the day, the environment of the session, the number of horses in the same area, the number of people in the same area, the age of the client, type of disability, the balance of the client (therapeutic riding), and mounting attributes needed.

Characteristics of the subject included sex, age, education, and work experience in this field. The questions about the horse and the horse-riding instructor/coach were to be filled in once, with the questions about the session each session.

### 2.2. Evaluation Exercise to Establish Behavioural Thresholds in EAS Sessions

Simultaneously to the field study, experts were asked to establish thresholds for all 21 behaviours within the “frequently observed” category. The selected equine and field experts possessed a combination of knowledge and experience in both equine behaviour and EASs. Those whose expertise focused more on equine behaviour were also expected to have a strong understanding of horse welfare. The expert evaluation involved assigning scores to predefined cases of virtual scenarios, similar as was done by the authors of [57] following the methodology established in the Welfare Quality project [58].

To determine the upper and lower thresholds for each behaviour, experts independently evaluated a dataset of 21 behaviours, each represented by 11 fictional sessions with specified prevalence rates. Each expert received a standardized set of cases, each representing a horse’s behaviour across 10 sessions (e.g., horse 1 never displayed “ears forward” more than 10% of the session time, horse 2 displayed “ears forward” more than 10% of the session time in one out of 10 sessions, horse 3 in two out of 10 sessions, horse 4 in three out of 10 sessions until horse 10 in 10 out of 10 sessions). In line with the methodology developed in the Welfare Quality project, and executed in breeding horses by the authors of [59], experts evaluated each case using a scale from 1 to 100, where 1 indicated the worst possible scenario for the horse’s welfare, 100 presented the ideal, and 50 denoted a neutral state. Scores above 80 reflected excellent behaviour, while those below 20 were considered unacceptable [58,59]. The definition of each behaviour was provided (as outlined in the ethogram, Table 1) and accompanied by short video clips to ensure consistent understanding among experts. Experts were instructed to assign a score of 50 to any of the 11 cases within a behaviour when they were unable to determine whether it should be classified as unacceptable, neutral, or excellent. To prevent the experts from influencing one another, they were seated at separate tables and discouraged from speaking throughout the evaluation. Experts recorded their scores on paper, which were subsequently entered into Excel (Office 365).

### 2.3. Data Analysis

#### 2.3.1. Equine Personality Profiles

The first part of the analysis focused on the equine personality profiles. These were determined using the method described by Ijichi, Collins, Creighton, and Elwood [56], yielding scores for agreeableness, extroversion, and neuroticism. First, the normality of the behavioural scores was tested using a SaphiroWilk test with an Alpha of 0.05. A two-way ANOVA was then conducted to identify differences in personality profiles both within and between horses used for equine-assisted coaching and therapy.

#### 2.3.2. Observed Behaviours Versus Expert’s Thresholds

The second part of the analysis focused on comparing the behaviours observed during the field study with the thresholds established by the experts. To begin with, behavioural scores assigned after each session were converted into percentages of the total sessions per horse throughout the study, reflecting the occurrence of the two categories (observed occasionally and observed frequently) over a two-month period.

Next the expert’s thresholds for equine behaviour were compared with the category of frequently observed behaviour in the field study. Experts were asked to give thresholds for all behavioural scores to determine whether a given percentage of frequently observed behaviour represented an excellent, neutral, or unacceptable situation. Twenty-eight experts participated in this classroom desk-based exercise. For each behaviour, an expert’s results were excluded from further analysis if (1) they assigned a score of 50 to all 11 cases (as instructed when unable to determine a score), (2) their scoring lacked linearity (visual analysis), or (3) if their scoring direction contradicted that of 75% or more of the other experts. This percentage was selected as it aligns with methodologies where a 75% agreement threshold is used to assess consensus [54].

#### 2.3.3. Impact of External Factors

The third part of the analysis focused on examining the impact of external factors on behavioural responses. To start with, Principal Component Analysis (PCA) was applied to identify underlying components in the behavioural responses of horses during equine-assisted coaching and therapy, with an orthogonal rotation used to better distinguish these components. The selection of the number of components was done using the Kaiser rule (eigenvalues > 1) and by examining the “elbow” of the scree plot. Furthermore, the threshold for explained variance was set at 60%. In the case that these selection methods gave different outcomes, the loading matrices of these solutions were compared, and a selection was made based on the cleanest (fewest cross-loadings) loading matrix. Subsequently, multiple linear regression was conducted to assess the influence of horse-related, management-related, and session-related variables on the PCA component scores. Information criteria were applied to identify the most significant and influential variables. The analysis was performed separately for coaching and therapy, given the distinct nature of each activity. Researchers labelled component labels based on behavioural variable loadings in consultation with field experts.

All analyses were performed using the statistical programs JASP and R. For all analyses, values of *p* < 0.05 were considered statistically significant.

## 3. Results

Between October 2022 and July 2023, a total of 830 EAS sessions were monitored and scored by 50 independent trained horse-riding instructors and coaches.

### 3.1. Session Characteristics

This study examined 432 equine-assisted coaching sessions and 398 therapeutic riding sessions. On average, a horse in the equine-assisted coaching program participated in four sessions over a two-month period, with the maximum participation reaching 10 sessions, whereas a therapeutic riding horse was involved in an average of 31 sessions, peaking at 61 sessions within the same timeframe. Additionally, there was a difference in session lengths: equine-assisted coaching varied from 15 to 60 min per session, where 25% of the sessions were between 15 and 30 min, 27% between 30 and 45 min and 28% between 45 and 60 min. Therapeutic riding sessions were, with 73% of the sessions, consistently between 30 and 45 min.

Of the coaching clients, 63% were familiar with their coaching horse, while 37% were not. The age of the coaching clients ranged from 4 years to over 50 years, with the majority (24%) falling in the 4 to 12-year age category. Additionally, 71% of coaching clients did not have a psychological condition, whereas 29% did. In contrast, 96% of clients involved in therapeutic riding sessions were familiar with their horse, and only 4% were not. The age range of these clients varied from under 18 years to over 60 years. Among the therapeutic riding clients, 64% had a cognitive disability, 18% had a physical disability, and 18% had a combination of both cognitive and physical disabilities.

### 3.2. Characteristics of Riding Instructor or Coach

This study included a total of 13 horse-riding instructors, of whom only 8 documented their characteristics. For the equine-assisted coaching portion of the study, 35 coaches participated, with 32 of them recording their characteristics. All coaches and instructors involved in the study were female. Seventy-five percent of the coaches were over 40 years old, while 63% of the therapeutic riding instructors were above the age of 40.

Thirty-one of the 32 coaches had received formal education in coaching, while 6 out of 8 therapeutic riding instructors had completed training to become an instructor. Forty-one percent of the coaches had at least five years of experience in their field, compared to 67% of the therapeutic riding instructors.

### 3.3. Characteristics of the Horses and Their Management

Data were collected on 85 horses engaged in equine-assisted coaching (40 mares, 44 geldings, 1 stallion) and 13 horses used in therapeutic riding (6 mares, 7 geldings). The horses used for equine-assisted coaching ranged in age from 2 to 28 years, with an average age of 14. The horses used in therapeutic riding lessons ranged in age from 4 to 27 years, with an average age of 14. In both equine-assisted coaching and therapeutic riding, 20% of the horses had a diagnosed health problem, such as arthritis, kissing spines, Cushing’s Disease (PPID) or Polysaccharide Storage Myopathy (PSSM).

Differences were found in the housing and feeding management of horses used in equine-assisted coaching compared to those used in therapeutic riding (see Table 2). Seventy-five percent of the therapeutic riding horses were mainly housed in individual stalls, whereas only 27% of the horses used for equine-assisted coaching were housed in this way. Horses used for equine-assisted coaching were primarily kept in group housing outdoors. Although roughly half of the horses in both groups had *ad libitum* access to roughage (including pasture), between 30.4 and 32.9% of the horses experienced periods lasting more than six consecutive hours without access to roughage. Horses involved in equine-assisted coaching were more often kept in pasture or paddock environments than those used in therapeutic riding.

**Figure 1:**
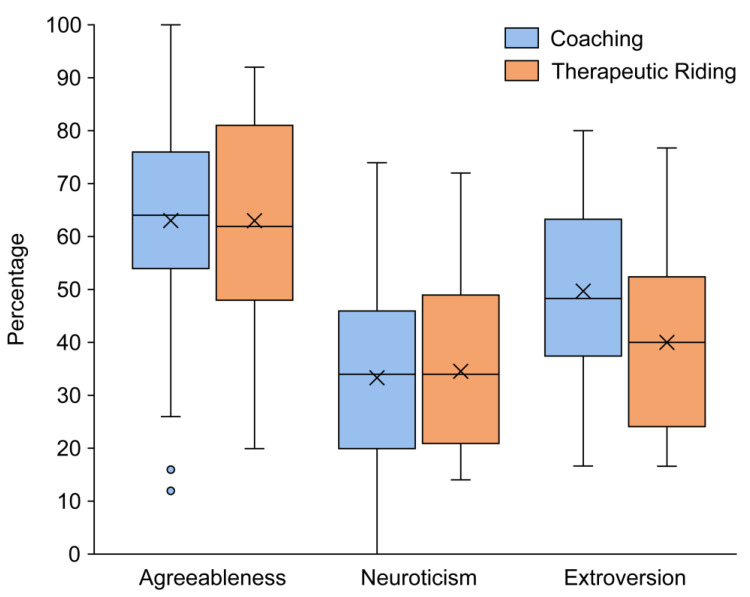
Distribution of percentage scores for Agreeableness, Neuroticism, and Extroversion observed during Coaching (blue) and Therapeutic Riding (orange) sessions. The boxplots display the median (horizontal line), interquartile range (box), whiskers (range within 1.5 times the interquartile range), and outliers (dots). Mean scores are marked with X.

The personality of horses encompasses all the unique traits, behaviours, emotions, and thoughts that are characteristic of an individual [60]. These personality traits are stable over time and across different situations [61]. The personality of the horses in this study was profiled using an equine personality survey [56]. The results of the two-way ANOVA indicate that the personality profiles of horses used in equine-assisted coaching do not significantly differ from those of horses used in therapeutic riding (*F*(1) = 0.901, *p* = 0.343). This suggests that the same type of horse is selected for both types of services. However, within each service, there is a significant difference across all three personality traits (*F*(2) = 32.186, *p* < 0.001). No interaction effect was found between personality traits and the type of EAS the horses participated in (*F*(2) = 1.346, *p* = 0.262). On average, horses used for equine-assisted coaching and therapeutic riding are predominantly agreeable (63% ± 17.63), moderately extraverted (48% ± 16.90), and score relatively low on neuroticism (33% ± 17.57) (see Figure 1).

### 3.4. Horses’ Behaviour

After each EAS session, horses were evaluated on 21 specific behaviours (see Table 1 for the behavioural ethogram).

In 65% of the sessions, horses involved in equine-assisted coaching frequently displayed a relaxed tail. In 44% of the sessions, the horses frequently had their ears forward, and, in 42% of the sessions, they frequently maintained a low head and neck position. Meanwhile, behaviours such as blowing, snapping and biting, and rolling occurred in only 15%, 18%, and 20% of all sessions, respectively (see Table 3).

During therapeutic riding lessons, horses frequently displayed a relaxed tail in 87% of the sessions. Additionally, in 54% of the sessions, the horses were frequently flicking their ears (ears active), and, in 50% of the sessions, their ears were frequently forward. Rolling and blowing were not observed at all, while hind leg resting and self-grooming were noted in only 1% and 3% of the sessions, respectively (see Table 4).

### 3.5. Upper and Lower Thresholds for Behaviours Observed in EAS Sessions

Twenty-eight experts specializing in horse behaviour, welfare, health, and equine-assisted interventions, with a minimum of five years’ experience in the respective fields, independently assessed 11 fictional cases for each selected behaviour using an absolute evaluation scale from 1 to 100. This method determined the upper and lower thresholds for the occurrence of each specific behaviour displayed frequently by the horses during a 45 min session (see Table 5). Results show that it was unacceptable if horses had their ears forward frequently in fewer than 45% of the sessions, while it was deemed excellent if they had their ears forward frequently in more than 87% of the sessions. Similarly, it was considered unacceptable if horses had their ears flattened frequently in more than 43% of the sessions, and excellent if they had their ears flattened frequently in less than 10% of the sessions.

**Figure 2:**
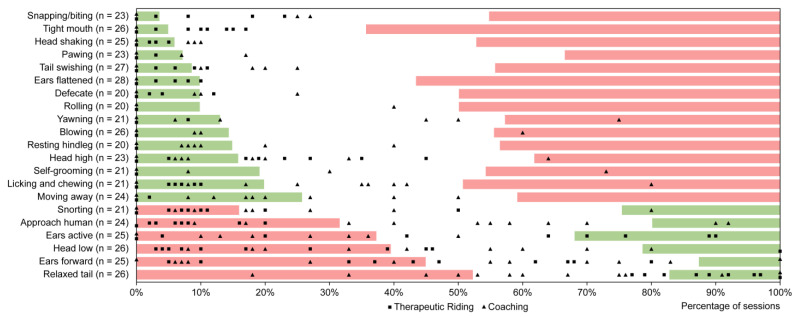
Presentation of the comparison between the current status of horses displaying specific behaviours during field study sessions and the upper and lower thresholds established by 28 experts (where ‘n’ denotes the number of experts involved in setting the threshold for each behaviour). The percentages on the horizontal axis represent the proportion of sessions within a given time period. Each square dot represents a horse used in therapeutic riding, while each triangular dot represents a horse used in equine-assisted coaching. The behaviours are arranged according to their lower thresholds. A green–white–red ordering indicates behaviour associated with a negative affective state, whereas a red–white–green ordering indicates behaviour linked to a positive affective state.

To evaluate the current status of behaviours observed by professionals during sessions, the field study data were compared with the established thresholds (see Figure 2). In Figure 2, behaviours are arranged according to their lower thresholds. A green–white–red ordering indicates behaviours associated with a negative affective state, whereas a red–white–green ordering represents behaviour linked to a positive affective state.

The comparison between the thresholds established in the expert consultation exercise and the data gathered from the field study revealed distinct patterns for behaviours associated with affective states. Behaviours identified by the experts as indicative of a negative affective state (considered excellent when observed in only a small number of sessions, as shown in Figure 2, from snapping/biting to moving away) were predominantly within the excellent (green) or neutral (white) range. For instance, a frequently observed tight mouth was considered unacceptable if present in more than 36% of sessions and excellent if observed frequently in fewer than 5% of sessions (Table 5 and Figure 2 green range). In the field study, horses frequently exhibited a tight mouth within the green and white areas, indicating this behaviour was excellent or neutral, respectively.

In contrast, behaviours identified by the experts as indicative of a positive affective state (e.g., approaching a human, ears active, head low, ears forward, and a relaxed tail) were observed in the field study with percentages spanning both unacceptable and excellent ranges. For example, a significant proportion of horses were observed with their ears forward frequently in less than 45% of the sessions (Table 5 and Figure 2, red range), which experts classified as unacceptable.

### 3.6. Affective States

A Principal Component Analysis identified six components with eigenvalue above 1 explaining 63 percentage of the total variation. The components were labelled as comfortable, tense, release tension, restless, agitated, and detached (see Table 6). The behaviour “head high” did not make a significant contribution to any of the identified components.

**Figure 3:**
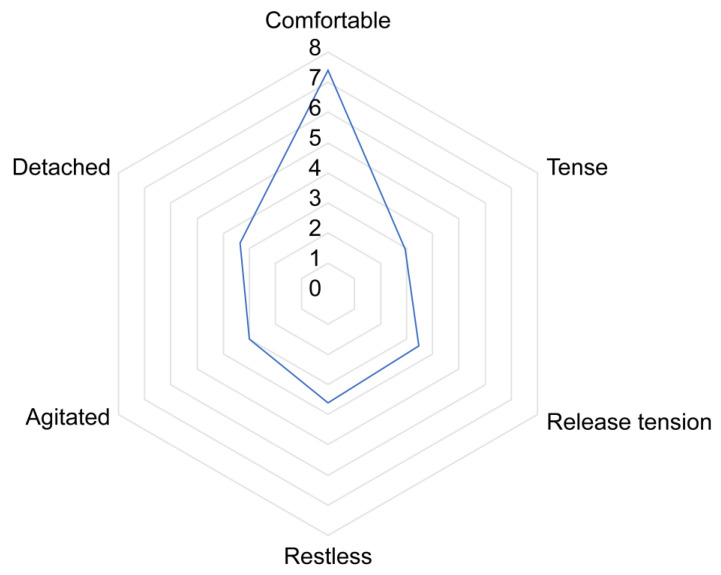
Radar chart illustrating scores for affective states during sessions, including Comfortable, Tense, Release Tension, Restless, Agitated, and Detached. Scores range from 0 to 8, with higher values indicating a stronger presence of the corresponding state. The chart provides a visual summary of the affected states displayed by horses during 432 equine-assisted coaching sessions.

For easier interpretation, the scores were rescaled to a 1–10 scale, with 1 indicating “not at all” and 10 indicating “all the time”. The average (SDEV) scores for the components were “comfortable”: 7.38 (1.78), “tense”: 2.90 (1.64), “release tension”: 3.44 (1.58), “restless”: 3.59 (1.72), “agitated”: 3.00 (1.88), and “detached”: 3.42 (2.20). The affective state labelled as “comfortable”, characterized by behaviours such as ears forward, head low, ears active and a relaxed tail, was the most predominantly displayed state by horses during equine-assisted coaching sessions (Figure 3).

The PCA regarding therapeutic riding sessions revealed four components with an eigenvalue above 1, explaining 72% of the total variation. The components were labelled as “building tension”, “agitated”, “release tension”, and “comfortable” (see Table 7). The following behaviours did not have a significant contribution to any of the components: relaxed tail, head low, snort, approach human, defecate, head shaking, pawing, yawning, rolling and self-grooming.

**Figure 4:**
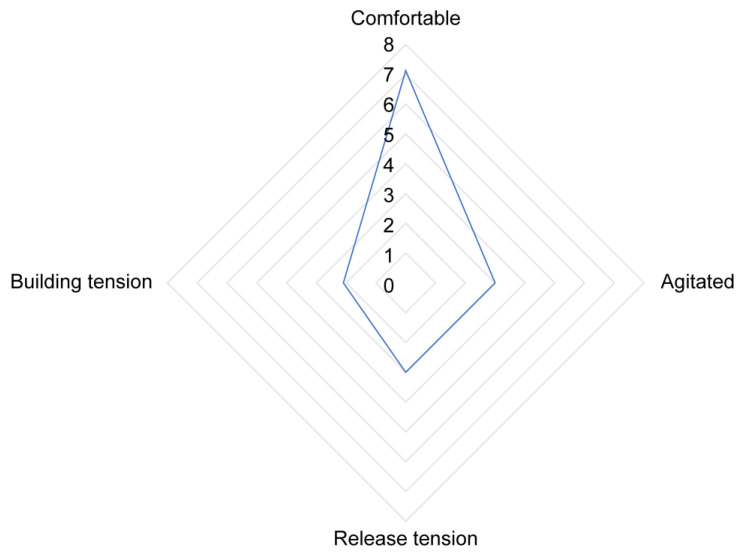
Radar chart illustrating scores for affective states during sessions, including Comfortable, Agitated, Release Tension and Building Tension. Scores range from 0 to 8, with higher values indicating a stronger presence of the corresponding state. The chart provides a visual summary of the affected states displayed by horses during 398 therapeutic riding sessions.

Scores were rescaled on a scale of 1 to 10. The average (SDEV) scores for the components were “Building tension”: 2.16 (1.29), “Agitated”: 3.07 (2.15), “Release tension”: 3.14 (2.30), and “Comfortable”: 7.15 (2.64). The affective state labelled as “comfortable”, here characterized by behaviours such as ears forward and ears active, was the most predominantly displayed state by horses during therapeutic horse-riding sessions (see Figure 4).

### 3.7. Association Between Horses’ Affective States and Horse, Session, and Management Characteristics

The data obtained from the field study did not show any association between the age, sex, height, or breed type of the horse and their affective states, and whether for horses used in equine-assisted coaching or therapeutic riding. On the other hand, the personality of the horse was associated with certain affective states. Horses used in equine-assisted coaching that were characterized as more extroverted were associated with a higher percentage of comfortable states (*t*(203) = 3.524; *p* < 0.001). Conversely, horses characterized as less agreeable were associated with more tense states (*t*(324) = 2.065; *p* < 0.05). Horses used in therapeutic riding that were characterized as more extroverted were associated with a higher percentage of building tension (*t*(293) = 2.865; *p* < 0.01). On the other hand, horses characterized as more agreeable were associated with a greater number of comfortable states (*t*(276) = 2.137; *p* < 0.01).

The results revealed significant associations between the affective states of horses and known clinical problems. Among horses used in equine-assisted coaching, 21% had clinical problems and were found to be significantly less comfortable (*t*(225) = −3.282; *p* < 0.01), more agitated (*t*(265) = 3.357; *p* < 0.001), and less detached (*t*(264) = −3.429; *p* < 0.001). However, although 23% of horses in this study used in therapeutic riding also had clinical problems, no significant relationship was found between these issues and their affective states.

The length of the coaching or therapeutic riding session was not associated with the affective states horses displayed during the sessions. During equine-assisted coaching sessions, horses exhibited fewer comfortable states when other horses were present in the same environment (*t*(203) = −2.597; *p* < 0.01). Additionally, when more people were present, the horses appeared more detached (*t*(264) =2.417 *p* < 0.05). In therapeutic riding sessions, having fewer horses in the same environment was associated with increased tension (*t*(303) = 2.389; *p* < 0.01). Furthermore, more people around the horses guiding the client was associated with both increased tension (*t*(303) = 6.182; *p* < 0.001) and tension release (*t*(303) = 2.380; *p* < 0.05). Conversely, when more people were present guiding the horse during the session, the horses were found to be more agitated (*t*(291) = 2.696; *p* < 0.01) and less comfortable (*t*(276) = 3.503; *p* < 0.001). A higher level of education attained by the instructor was significantly associated with the horses displaying less tension buildup (*t*(293) = 9.610; *p* < 0.001) and reduced agitation (*t*(286) = −12.36; *p* < 0.001).

In therapeutic riding, various mounting aids, such as a mounting platform and a mounting stool, were used. The results revealed a significant negative association between the use of a mounting stool and the display of a comfortable state by the horse (*t*(276) = −3.692; *p* < 0.001). Additionally, when clients had an unbalanced seat (either to one side or leaning backward), the horses were significantly less comfortable (*t*(203) = 2.016; *p* < 0.01 and *t*(286) = 3.711; *p* < 0.001, respectively).

Horse management practices were significantly linked to the horses’ affective states during sessions. In equine-assisted coaching, horses kept in a pasture or paddock for 24 h a day exhibited notably fewer behaviours associated with comfort (*t*(203) = −2.309; *p* < 0.01) and tension release (*t*(324) = −3.394; *p* < 0.001). Conversely, horses in this setting that were kept in a paddock or pasture for less than 6 h a day showed significantly more behaviours indicative of a comfortable affective state (*t*(203) = 2.016; *p* < 0.01) but also exhibited increased restlessness (*t*(395) = 8.556; *p* < 0.001). Horses in therapeutic riding settings that were kept in paddocks or pastures for 24 h a day when not working showed significantly more agitation during sessions (*t*(286) = 4.219; *p* < 0.001), whereas horses that spent less than six hours a day in a paddock or pasture were significantly less agitated (*t*(286) = −4.371; *p* < 0.001).

For horses used in equine-assisted coaching, spending more than six hours without access to roughage was negatively correlated with a comfortable state (*t*(203) = −1.994; *p* < 0.05). Additionally, *ad libitum* roughage/grazing was negatively associated with a comfortable state in horses used for equine-assisted coaching (*t*(203) = −4.340; *p* < 0.001) but positively associated with a comfortable state in therapeutic riding horses (*t*(276) = 13.444; *p* < 0.001). In therapeutic riding horses, spending more than six hours without access to roughage was negatively associated with agitation during sessions (*t*(286) = −3.983; *p* < 0.001).

## 4. Discussion

The findings presented in this paper are the first to document both positive and negative equine affective states during equine-assisted coaching and therapeutic riding sessions, as observed by trained practitioners. The results are drawn from a large-scale population study conducted over an extended period, offering insights into how horses perceive the sessions, and the components possibly associated with their affective states. Additionally, an ethical evaluation of fictional examples has established thresholds for behaviours with both positive and negative valence exhibited by the horses during the sessions.

### 4.1. Session, Horse, and Management Characteristics

Consistent with findings from other studies [62,63,64], the number of weekly sessions offered by EAS providers varied significantly among practitioners. From the horses’ perspective, the number of sessions per horse in this study also showed considerable variation: for equine-assisted coaching, this ranged from 4 to 10 sessions over a two-month period. Data on the size of the EAS providers or the number of horses they owned was not collected. Most subjects worked part-time in equine-assisted coaching, used one or two horses, and the horses were often used for additional activities beyond coaching. This indicates that the majority of subjects likely represented relatively small companies. Offering just one session every two weeks appears to be a relatively low frequency for generating sufficient income to sustain the business. In contrast, therapeutic riding programs delivered 32 sessions over a two-month period, equating to 3 to 4 sessions per week. This aligns with the finding by Watson et al. [65], who reported that horses in therapeutic riding programs were utilized four days a week. Furthermore, 77% of riding instructors in therapeutic riding used multiple horses, suggesting participation from larger companies.

In Seery and Wells [63], equine-assisted coaching sessions typically lasted between 30 and 60 min; however, in this study, the duration ranged from 15 to 60 min. In contrast, the length of therapeutic riding sessions was more consistent, averaging between 30 and 45 min, which is similar to what was reported in the study of Watson, Davis, Splan, and Porr [65]. The difference in session length variability reflects the nature of the activities, with therapeutic riding being a more standardized practice typically offered in a regular riding school, where it must align with the horses’ daily schedule.

The profile of the coaches and riding instructors concurs with that found in other studies. In this study, they were all females, with the vast majority over 40 years of age. In Seery and Wells’ [63] study the vast majority of the EAS practitioners were female and over 50 years of age. Practitioners working with horses in health services may benefit from a diverse and specialised range of skills. This may include strong interpersonal and service skills, extensive knowledge of disabilities, proven expertise in equine care (with an awareness that horses are able to experience affective states), and the ability to effectively manage interactions between horses and humans—all while prioritizing overall equine welfare. These skills are crucial because working with horses, whether mounted or on the ground, carries a significantly increased risk of injury [66], which is often overlooked in EAS research [63]. This is especially important given that the client base often includes vulnerable individuals, where obtaining explicit consent regarding working in a safe environment can be challenging. The skills needed to ensure this safe environment are primarily equine-related. In an earlier study, it was found that the type of EAS provided was also linked to whether practitioners held an equine-related qualification. Those offering learning or riding services were more likely to possess such qualifications [63]. Unfortunately, that study collected information on the educational background of the coaches and instructors but did not explicitly differentiate between equine-focused or client-oriented education. In this study, nearly all coaches and instructors had completed a coaching or instructing educational program, with nearly 40% of the coaches and almost all instructors having over five years of experience in the field. While this experience might help compensate for the lack of formal equine-specific qualifications, research indicates that extensive experience alone does not necessarily enhance the ability to accurately identify equine behaviours or assess horse welfare [67,68]—both of which are crucial for ensuring human safety.

The present study found that horses in equine-assisted coaching had a broader age range than those used in therapeutic riding, though the majority in both groups were between 10 and 19 years old. Specifically, 20% of the horses in equine-assisted coaching were younger than 10 years, and another 20% were older than 19 years. In contrast, only 8% of therapeutic riding horses were over 20 years old, and 16% were under 10. These findings align with Lerch et al. [69], who reported that horses used in EAS averaged 14.3 years of age, but differ from Watson, Davis, Splan, and Porr [65], where horses in therapeutic riding programs were most commonly aged between 16 and 20 years, with additional groups over 20 and between 11 and 15 years. However, it is important to note that the study involved a relatively small number of horses in therapeutic riding sessions, with only 13 participating. Similar to other studies, most horses used in EAS are either geldings or mares. In this study, the number of mares and geldings was roughly equal, while in Watson, Davis, Splan, and Porr [65], about two-thirds of the horses were geldings and, in Lerch, Cirulli, Rochais, Lesimple, Guilbaud, Contalbrigo, Borgi, Grandgeorge, and Hausberger [69], 54% mares and 46% geldings. The differences in the number of mares and geldings used for EAS in these studies may be explained by cultural factors. While the first study was conducted in the United States, the second was carried out in France, Italy, and Ireland. In the US, geldings have historically been preferred due to their role in Western riding and the ranching industry, where temperament and obedience are crucial. This may explain their popularity in EAS programs. In Europe, both mares and geldings have traditionally been used in sport and work, leading to more familiarity with working with both sexes. These variations highlight the influence of regional equestrian traditions on horse selection for EAS, demonstrating that both mares and geldings can successfully adapt to these roles.

While most practitioners claim to select horses based on personality traits, only a few studies have explored this practice [70,71,72]. From a safety perspective, it could be argued that practitioners tend to select horses with low reactivity to minimize risks for clients. However, for effective interaction with clients, it might be beneficial to choose horses with at least above-average reactivity towards humans. Furthermore, Anderson, Friend, Evans, and Bushong [70] found that therapeutic riding instructors struggled to reach consensus on the personalities of their centre’s horses based on reactivity scores. In Minero, Zucca, and Canali [71], who examined four horses, it was concluded that horses used in EAS sessions were not significantly more reactive in restraint and startle situations. In the present study, horse personalities were assessed through the Equine Personality Test, a survey method [56]. Ijichi, Collins, Creighton, and Elwood [56] validated the survey method by comparing it with behavioural test data, thereby demonstrating its accuracy in predicting behaviour and its potential as an alternative to objective personality measurements. In this study, both horses used in equine-assisted coaching and those in therapeutic riding scored highest for the trait of agreeableness (63%), followed by extraversion (48%). The lowest scores were observed for neuroticism (33%). Because of a technical issue with the application used to collect data in this study, the trait of gregariousness towards people and horses, which is part of the Equine Personality Test by Ijichi, Collins, Creighton, and Elwood [56], was not included. The personality profile of EAS horses found in this study align with the essential characteristics needed for their roles in EAS. EAS horses must be adaptable across various activities and suitable for different clients. This necessitates a certain degree of agreeableness. The average level of extraversion allows these horses to communicate their affective states, which enhances the potential for communication between the horse and the client. The low scores on neuroticism can be explained by the fact that excessive fear and stress responses are likely undesirable concerning client safety. In line with these results, DeBoer [73] showed in her study based on a 36-question survey that equine-assisted service professionals considered “curious”, “tolerant”, “calm”, “sociable”, and “gentle” to be the most desirable personality traits for horses working in equine-facilitated mental health, while traits like “fearful”, “unpredictable”, “anxious”, “excitable”, and “solitary” were considered the least desirable. Although those results may suggest that EAS horses are selected based on a specific personality profile, previous studies found no significant differences in personality between EAS horses and those used for other purposes. Brubaker, Schroeder, Sherwood, Stroud, and Udell [72] found no significant differences in personality test results between horses selected for EAS and those not selected. However, during a sociability test in their study, non-selected horses exhibited significantly more affiliative behaviours towards a familiar person than selected horses. Also, Minero et al. (2006) reported in their study that therapeutic riding horses did not show less reactivity to new stimuli than jumping horses. Taken together, those results may indicate that the personality profile that was found in this current study might be a reflection of that of the average horse population and might be advantageous across various disciplines where there is close horse–human interaction.

In addition to the limited data on equine characteristics in EAS, such as details about their housing, management, and prior experiences, there is a need for more comprehensive information specifically on the health of horses involved in these activities. These data are crucial for assessing and monitoring how participation in EAS may affect horse welfare. Moreover, it is well established that a horse’s suboptimal health condition can negatively impact its behaviour toward humans [74]. This study examined various preventive health management practices and the presence of any diagnosed health issues. It was found that over 20% of the horses in the study had a diagnosed health condition. To the best of our knowledge, there have been no recent studies on health issues in the overall equine population in the Netherlands. Consequently, it remains uncertain whether the percentages we observed correspond with those in the broader horse population. However, earlier studies and research focused on specific segments of the horse population have reported similar percentages. A study by Visser et al. [75] indicated that approximately 20% of horses experienced locomotion problems, while nearly 30% reported some form of back pain. Additionally, research on senior horses (aged over 15 years) in the UK and the US has shown a high prevalence of owner-reported medical conditions [76,77]. The notable percentage of horses with diagnosed health issues in our study raises concerns, particularly given that equine health is emphasized as a crucial topic in the foundational training for practitioners in EAS [63]. This highlights the need to carefully consider the background and physical well-being of horses selected for EAS, as their prior experiences and health status can significantly influence their suitability for such work. Practitioners often mention that many EAS horses have had one or more previous careers, with very few—if any—being specifically trained for EAS work from a young age. In EAS, facility management decisions are made by the manager, resulting in a wide range of living conditions for equids, including differences in housing, feeding, and social environments. Key factors known to significantly impact horse welfare include whether they are housed indoors or outdoors and whether they are kept individually or in groups; see, e.g., [78,79,80]. In this study, horses used for equine-assisted coaching were most commonly housed outdoors in groups (50% of the horses in this category), whereas those involved in therapeutic riding were kept indoors in individual stalls (75.0% of the therapeutic riding horses in this study). This difference is anticipated due to the varying facilities, as the horses used for therapeutic riding were also utilized for regular lessons and therefore housed in riding schools. Providing *ad libitum* access to roughage is linked to a reduction in stereotypic behaviours and colic, as well as an increase in positive interactions with both conspecifics and humans [81,82]. Horses should not go without roughage for longer than 4 to 6 h. Extended periods without roughage can lead to serious health issues, including an increased risk of gastric ulcers and colic. This is supported by research, such as the work of Hudson et al. [83], who established a connection between feeding management and the risk of colic in horses. The results of the present study indicate that, while more than half of the horses had *ad libitum* access to roughage, nearly one-third were still without roughage for more than 6 consecutive hours a day. This is a substantial percentage, highlighting the need to spread awareness of this issue within the EAS sector.

### 4.2. Behavioural Responses

Earlier studies have employed various methods to assess behaviour and welfare during sessions, with a common approach being the use of an overall behavioural score derived from summing individual stress-related behaviours observed over a defined period of time [23,42,53,84]. More recently, Rankins, McKeever, and Malinowski [54] applied qualitative behaviour analysis as a method for assessing horse welfare. In the present study, retrospective scoring of behavioural categories was selected as the most practical and feasible approach, both for conducting the research and for its potential application in real-world practice. However, this choice represents a limitation, as it may influence the accuracy and consistency of behavioural assessments. While no data exist on the accuracy of this method, a pilot study was conducted to assess its reliability. In this pilot, 24 sessions were videotaped and analysed using The Observer software (Noldus Observer XT15), applying the same behavioural categories used in this study. The comparison between trained subjects’ retrospective scoring and video-based scoring in The Observer software showed agreement levels ranging from 33.5% (chewing and licking) to 100% (rolling). In the study by Czycholl et al. [85], which examined rater agreement for horse welfare indicators, agreement thresholds were based on Plesch et al. [86], where a kappa value of 0.4 was considered acceptable and 0.6 good. These findings indicate that while variability is present, trained subjects may be able to provide reasonably reliable retrospective assessments of horse behaviour when in using a structured ethogram and standardized scoring criteria. However, further research is required to enhance the reliability of this method for practical applications.

Rudd, Wheeler, Pasiuk, and Schroeder [67] also investigated equine behaviour in the context of EAS, incorporating several behaviours that overlap with those assessed in the present study. Their research focused on volunteers’ perceptions of horse behaviour, highlighting key behavioural indicators relevant to both equine welfare and human–horse interactions. The inclusion of similar behavioural observations in both studies underscores the importance of evaluating equine responses during EAS sessions to ensure not only the welfare of the horse but also the effectiveness of the service. In the present study, behaviours were carefully selected to represent the horse’s subjective emotional experiences [87], characterized by both valence (e.g., positive or negative, pleasant or unpleasant) and arousal/intensity level (e.g., contentment versus excitement) [29,88]. While some behaviours arguably indicate a positive or negative valence (ears forward and ears flattened, respectively), others can only be understood when considered alongside a combination of different behaviours. For example, nibbling combined with licking and ears forward is considered a positive sequence of behaviours towards humans, whereas nibbling accompanied by actual bites and ears flattened is viewed as a negative sequence of behaviours towards humans [69]. To capture these categories, a rubric was utilized for data collection, recording either the duration (percentage of session time) or the frequency (number of occurrences within a session). It was hypothesized that a rubric, rather than a checklist or a Likert scale without detailed descriptions, would result in a valid and reliable assessment tool that is both practical for use in the field and aligns with recommended EAS equine assessment criteria [89,90]. It was expected that the session lengths in equine-assisted coaching would be less variable than they ultimately were. As a result, the behaviours recorded as frequencies per session may have been either under- or overestimated in the study. Due to the large sample size in the study, it was anticipated that the effect would be minimal; therefore, the scores were not adjusted for session length.

The ear positions—forward and active—were frequently observed in both equine-assisted coaching and therapeutic riding sessions. Since previous studies have focused on stress-related behaviours to assess welfare during EAS, there are, to our knowledge, no studies available for comparison regarding these percentages for ear positions. Ears forward in horses typically indicate that the horse is attentive, curious, or interested in something in its environment. This ear position often suggests that the horse is focused on an object, person, or sound in front of them, and it is generally a sign of alertness and engagement rather than stress or fear [91]. Actively flicking and rotating ears in horses typically indicate that the horse is attentively listening to its environment, scanning for sounds, or processing multiple stimuli simultaneously [92]. This behaviour reflects the horse’s awareness and engagement with its surroundings, as horses can rotate their ears independently to better locate the source of sounds. From a welfare perspective, ear rotation can be a sign of alertness and interest, rather than stress or discomfort, especially when combined with other relaxed body language cues. Flattening of the ears was observed in 23% of the equine-assisted coaching sessions and 33% of the therapeutic riding sessions. This ear position is viewed as a stress behaviour [41,43,49] and was the most common stress-related behaviour displayed during mount and dismount in the study of McDuffee, Carr, and Montelpare [23].

Tail positions, such as relaxed tail and tail swishing, were included as behavioural indicators in the study. In 65% of equine-assisted coaching sessions and 87% of therapeutic riding sessions, horses exhibited a relaxed tail carriage for at least 30% of the session duration. While tail swishing is widely recognized across studies as an indicator of stress [35,93,94], a relaxed (swinging) tail has primarily been described in studies focused on the natural behaviour of horses in the wild while foraging [91]. In the current study, tail swishing was recorded as a frequency behaviour, categorized as either not occurring, occurring occasionally (1 to 5 times per session), or occurring frequently (more than 5 times per session). This behaviour was not at all observed by the trained practitioners in 46% of the equine-assisted coaching sessions and 43% of the therapeutic riding sessions. However, in the study of McDuffee, Carr, and Montelpare [23] tail swishing was identified as the second most common behaviour observed during mounting and dismounting in therapeutic riding sessions.

Based on an ethical exercise where 28 experts evaluated the welfare of fictional sessions, the 21 behaviours selected for this study were identified as having either a positive or negative valence. Roughly two-thirds of the behaviours that were selected for the study were identified by the experts as having a negative valence, and one-third as having a positive valence. The latter category includes behaviours such as snorting, approaching a human, ears active, a low head position, ears forward, and a relaxed (swinging) tail. For behaviours with a negative valence, experts deemed it ideal when these behaviours were frequently displayed in less than 4% of sessions (such as snapping and biting) to less than 26% of sessions (such as moving away). Conversely, for behaviours with a positive valence, experts considered it ideal when these behaviours were frequently observed in at least 68% of sessions (such as ears active) to 87% of sessions (such as ears forward). When these upper and lower thresholds per behaviour were compared with the actual data from the field study, it became evident that most behaviours with a negative valence were observed at lower percentages than the threshold deemed unacceptable. However, for behaviours with a positive valence, a significant proportion fell below the lower threshold, indicating that these behaviours were observed in far fewer sessions than what experts considered acceptable. For example, horses frequently displaying ears forward (at least 10% of the session) were observed in too few equine-assisted coaching and therapy sessions. Experts considered it acceptable if this behaviour was exhibited in at least 45% of the sessions for a particular horse; however, in the field study, this was not the case for half of the horses. This could be attributed to several factors. First, there may be a genuine discrepancy between the desired minimum percentage of positive valence behaviours and what the horses actually exhibit in practice. Second, although practitioners were trained to accurately observe and score all 21 behaviours, there might have been a tendency to more readily recall behaviours with a negative valence over those with a positive valence. This could have resulted in lower reported percentages of positive valence behaviours in the field study data. This phenomenon is referred to as the “negativity bias” in the psychological literature [95]. The negativity bias refers to the tendency for negative events, experiences, or information to have a greater impact on an individual’s psychological state and processes than neutral or positive ones. This bias likely evolved as a survival mechanism, as being more attuned to potential threats (negative stimuli) would have had evolutionary advantages. In the context of observing animal behaviour, negativity bias can lead to an increased focus on and memory of behaviours that indicate potential danger, stress, or discomfort (negative valence), as these could signal a threat or a need for intervention. Positive behaviours, while important, may not trigger the same level of cognitive or emotional response, making them less memorable [95,96].

### 4.3. Affective States

While assessing specific behavioural responses allows for quantification, it offers only a limited understanding of the broader context [54]. Additionally, when behavioural indicators are evaluated in isolation, particularly those related to aggression, there is a risk of misdiagnosis [97]. To evaluate affective states throughout an entire session, Principal Component Analysis (PCA) was employed to identify which behavioural responses were correlated within the session. To the best of our knowledge, no studies have yet utilized PCA to analyse affective states in EAS. However, PCA has been shown to be a powerful tool in determining complex behavioural patterns and temperament traits in horses [31,60,98,99]. In the present study, PCA did not yield a straightforward model analogous to the valence–arousal circumplex with two factors (two axes). In equine-assisted coaching, six components were identified, while in therapeutic riding settings, four components were identified. The components identified explained 63% of the total variance in equine-assisted coaching sessions and 72.2% in therapeutic riding sessions. These results are consistent with previous studies, where explained variance ranged from 52.0% [98] to 84% [99]. The PCA revealed similar components between the equine-assisted coaching horses and the horses used for therapeutic riding, leading to them being labelled as representing the same affective states. In both types of assisted services, horses scored highest on the components labelled “comfortable.” This affective state is characterized by behaviours such as ears pointing forward and ears active, and in equine-assisted coaching, it also includes a low head position and a relaxed tail carriage. Additionally, it was observed that negative affective states, such as agitation, building tension, and tension, had consistently low average scores across the sessions. Based on these results, it was suggested that most of the horses in this study were in a positive affective state during these sessions. While some PCA components could be clearly interpreted as having a positive or negative valence, this was not true for all components. In equine-assisted coaching, the component labelled as “release tension” was associated with behaviours such as defecating, yawning, licking and chewing, pawing, and blowing. In therapeutic riding sessions, this component was characterized primarily by blowing and licking and chewing. While defecation (when not related to feeding) is typically interpreted as a stress-related behaviour [39,100], licking and chewing are often observed when horses are regaining from a stressful moment. It has been hypothesised that during this time, activation of the parasympathetic nervous system occurs, resulting in increased saliva production [101]. The component labelled as “restless” has positive loadings for approach human, moving away from human, snapping and biting, rolling, and pawing. Similar to the component labelled as “release tension”, some of these behaviours can be interpreted as having a negative valence (e.g., snapping and biting), while others are likely associated with a positive valence (e.g., approaching a human). Another interesting component appeared to be “detached”, characterized by hind leg rest and self-grooming. Apart from maintenance behaviour, self-grooming is a common displacement behaviour across various species, including mammals [102]. Displacement behaviours are actions that are out of context and usually occur when an animal experiences conflicting motivations, frustration, or stress. Self-grooming, in this context, is often seen when an animal is in an uncomfortable situation and is unsure how to respond and is thought to be a coping mechanism that helps the animal to manage stress or emotional conflict [103]. Further research is needed to determine whether the affective state labelled as “detached” may represent a form of displacement behaviour.

While practitioners often assert that horses used for therapeutic riding are typically chosen for their calm temperament [104], this has not been studied in detail. Furthermore, the personality characteristics of the horses have not been related to the affective states or related behaviours they display during sessions. In the present study, the personality traits of agreeableness and extroversion were found to be linked to the affective states displayed by horses during sessions. Extroverted horses were more likely to show comfortable affective states during equine-assisted coaching, while more agreeable horses demonstrated similar positive states in therapeutic riding programs. Given the distinct nature of these sessions, it is compelling that horses in equine-assisted coaching would exhibit more pronounced behaviours, which are likely more effective in fostering human–horse interaction. Additionally, horses with agreeable temperaments are seen as more secure and safe for therapeutic riding. Understanding that extroverted horses are prone to displaying comfortable and effective behaviours in equine-assisted coaching can assist practitioners in selecting horses with these traits for such sessions, potentially enhancing client engagement and interaction quality. Similarly, recognizing that agreeable horses are perceived as safer for therapeutic riding underscores their suitability for environments where client safety and calm, consistent interactions are crucial. This knowledge can inform decisions about horse selection for different therapeutic purposes, ensuring that horses’ natural tendencies align with the session’s objectives. In practice, this approach could enhance horse welfare by involving them in activities suited to their personalities. Additionally, it is likely to result in improved therapeutic outcomes for clients, as the horses’ behaviour would foster more effective human–animal interaction. This alignment could also reduce stress for the horses, further enhancing their well-being and effectiveness in therapeutic roles.

The health and soundness of horses are integral to the safety, effectiveness, and ethical integrity of EAS programs, directly influencing the well-being of both the horses and, presumably, the participants involved. It can be argued that horses that are healthy and sound are more likely to cope with the demands of the work without experiencing stress, pain, or injury. Moreover, healthy horses are less likely to exhibit unpredictable or unsafe behaviours that can arise from discomfort or pain. Soundness ensures that the horses can move and perform tasks reliably, reducing the risk of accidents during sessions [105]. And a horse that is unwell or uncomfortable may not be able to fully participate in interventions, diminishing therapeutic benefits for clients [20]. In the present study, data were collected on both preventive health management practices (e.g., vaccinations, farrier, deworming) and diagnosed health issues. In both equine-assisted coaching and therapeutic riding, a notable percentage of horses had diagnosed health issues (21% in equine-assisted coaching and 23% in therapeutic riding programs). However, a clear link between these health issues and the horses’ affective states was evident only in equine-assisted coaching. In this context, horses with diagnosed health problems showed significantly higher scores for negative affective states and significantly lower scores for positive affective states. With only 13 horses in the therapeutic riding group, it is possible that a relationship might have emerged if a larger number of horses had been included in the study. The study by Taschetto et al. [106] found that 72% of the 21 horses used in therapeutic riding were assessed as having a mild degree of lameness. As was stated earlier, maintaining horse health is not only essential for the welfare of the horses but also directly impacts the quality of the EAS. Practitioners should incorporate these insights into their care routines and session planning to ensure the welfare and safety of both horses and clients.

No relationship was found between the duration of the sessions and the affective states exhibited by horses in both equine-assisted coaching and therapeutic riding. This finding suggests that the session lengths used in this study (15 to 60 min for equine-assisted coaching and 30 to 45 min for therapeutic riding) did not have a negative impact on horse welfare. However, a notable factor that was significantly related to affective states in therapeutic riding was the client’s balance and seating position. Specifically, a client who sat unbalanced or leaned backward was significantly associated with the horse displaying an agitated state. To our knowledge this is the first study in which this relationship has been studied in EAS. Studies in regular horseback riding have shown a positive correlation between less skilled riders and an increase in stress-related behaviours in horses [107]. The fact that a client’s unbalanced seating position or backward lean is associated with the horse displaying an agitated state suggests that the physical stability and posture of the client are crucial factors in maintaining a calm and positive environment for the horse. It is recommended that practitioners prioritize training clients in proper balance and seating techniques before and during therapeutic riding sessions whenever possible. Additionally, continuous assessment of the client’s seating position during sessions should be a key focus. Using adaptive equipment, such as therapeutic saddles, and regularly checking this equipment can help reduce stress on the horse and enhance the overall quality of the session.

Only a small number of EAS studies investigate the housing conditions of the horses. Watson, Davis, Splan, and Porr’s [65] online survey, which was distributed to therapeutic riding centres in the US, showed that horses were most commonly kept on pasture when not in use. In the present study in the Netherlands, horse management practices were closely related to the horses’ affective states during the sessions, especially aspects connected to housing and feeding management. There appeared to be a direct relationship between the amount of time spent outside (in pasture or paddock) and the horses’ affective states during sessions. Horses kept outdoors 24 h a day tend to be less comfortable (in equine-assisted coaching sessions) and more agitated (in therapy sessions) than those that spend only part of the day outside. Furthermore, when horses have limited outdoor time (less than 6 h a day), they tend to be significantly more comfortable (in equine-assisted coaching sessions) and significantly less agitated (in therapeutic riding sessions). Compared to the experience of being outdoors in a pasture, both coaching and therapeutic riding sessions might be perceived as less enjoyable by horses. However, for horses kept stabled for extended periods, these sessions may offer a more engaging and pleasurable experience than simply standing in a stable. Further research is needed to gain a deeper understanding of the affective states identified in this study and their connection with housing and feeding practices.

Feeding management, particularly access to roughage, is closely connected to housing conditions. Consequently, it was not surprising to find that the amount of time horses had access to roughage was also related to their affective states during the sessions. When horses fast for more than 6 h at a time, it negatively impacts their health and welfare. Multiple studies have demonstrated a direct connection between prolonged fasting and the onset of colic and gastric ulcers [83]. Additionally, extended periods without foraging opportunities are associated with the development of stereotypic behaviours, particularly crib-biting and wind-sucking [108,109]. In the present study, it was observed that horses used for equine coaching that regularly fasted for more than 6 h at a time were less comfortable during the sessions, and likely outside them as well. However, it was found that horses in therapeutic riding sessions exhibited less agitated behaviour when their daily feeding management included more than six hours without roughage. It should be noted that the number of therapeutic riding horses in this study was limited compared to the number of horses used in equine assisted coaching. However, the data of the affective states of these horses (e.g., agitated) was based on a large number of sessions. One possible explanation for this finding could be the interaction between feeding and housing management. If these therapeutic riding horses, which were without roughage for more than six hours, were also kept in single stalls for most of the time, the chance to leave the stall, move around, or interact may have had a more significant impact on their affective states than the lack of foraging opportunities. However, these interpretations are speculative and require further research involving a larger sample of horses and EAS providers to validate.

## 5. Conclusions

This field study offers a comprehensive analysis of potential equine affective states during equine-assisted coaching and therapeutic riding, highlighting both positive and negative emotional responses in these settings. Trained practitioners scored equine behaviour, while an additional exercise with equine behaviour and field experts established thresholds for positive and negative valence behavioural indicators. These thresholds were then compared to field data to assess welfare levels in practice.

Using Principal Component Analysis (PCA), distinct affective states were identified for both equine-assisted coaching sessions as well as for therapeutic riding sessions. In most sessions, horses displayed an affective state that was labelled as comfortable. Factors influencing these affective states included horse health, session characteristics, management practices, and client posture. Horses without diagnosed health problems were more likely to display positive states in equine-assisted coaching, underscoring the need for robust health management to support welfare and safe interactions. In therapeutic riding, clients’ unbalanced seating was associated with increased horse agitation, highlighting the importance of rider skill in minimizing equine stress.

The study revealed that extroverted horses exhibited more positive affective states in coaching sessions, while agreeable horses showed similar positive states in therapeutic horse-riding settings. This suggests that matching a horse’s temperament with the specific type of session not only enhances the welfare and comfort of the horse but also the overall safety and effectiveness of the interaction, benefiting both the horse and the client. Management practices further influenced the horses’ affective states: horses with limited outdoor access displayed more relaxed behaviours during the sessions than those with unlimited outdoor access.

In summary, this study underscores the importance of health, suitable horse selection, and effective management practices in promoting positive affective states in equine-assisted services. These findings can be used to formulate guidelines for practitioners to align welfare practices with therapeutic goals, ensuring ethical standards, and improving client outcomes in equine-assisted programs.

## Figures and Tables

**Table 1:** Definition and scoring categories of behaviours included in the study, with reference to the sources on which the definitions were based.

Behaviour Label	Definition Used in the Study	Scoring Categories	Sources
Ears forward	Both ears are held simultaneously upright and directed forward for at least 3 s.	Absent: 0% of the session Occasionally: 1 to 10% of the session Frequently: more than 10% of the session	[33,34,35,36,37,38,39,40]
Ears flattened	Both ears are simultaneously directed downward and backward, forming an angle of less than 45 degrees with the neck.	Absent: 0 times during throughout the session Occasionally: 1 to 5 times throughout the session Frequently: more than 5 times throughout the session	[23,33,34,35,36,37,38,39,41,42,43]
Ears active	The horse’s ears move independently, shifting from front to back for at least 3 s, with the movement speed potentially varying between the left and right ear.	Absent: 0% of the session Occasionally: 1 to 10% of the session Frequently: more than 10% of the session	[35,36,39,40]
Head high	The nose or mouth is above an imaginary horizontal line drawn from the lowest point of the horse’s back and stays in this position for at least 3 s.	Absent: 0% of the session Occasionally: 1 to 10% of the session Frequently: more than 10% of the session	[23,35,37,38,41,42]
Head low	The nose or mouth is below an imaginary horizontal line drawn from the lowest point of the chest and remains in this position for at least 3 s.	Absent: 0% of the session Occasionally: 1 to 10% of the session Frequently: more than 10% of the session	[23,35,37,41,43]
Head shaking	The head moves up, down, and/or from side to side with a minimum displacement of 10 cm, and the movement is repeated at least twice.	Absent: 0 times throughout the session Occasionally: 1 to 5 times throughout the session Frequently: more than 5 times throughout the session	[23,34,35,37,39,41,42,43]
Relaxed tail	The tail moves in sync with the rhythmic motion of the horse’s gait.	Absent: 0% of the session Occasionally: 1 to 30% of the session Frequently: more than 30% of the session	[35]
Tail swishing	The tail moves forcefully from side to side, upward, downward, or in a circular motion. This behaviour is not recorded when it is a response to insects or due to imbalance associated with a forward gait transition.	Absent: 0 times during throughout the session Occasionally: 1 to 5 times throughout the session Frequently: more than 5 times throughout the session	[33,34,36,37,39,40,41,43,44]
Moving away	Within a 2-m radius, the horse’s head and neck move away from the client, increasing the distance between them.	Absent: 0 times throughout the session Occasionally: 1 to 2 times throughout the session Frequently: more than 2 times throughout the session	
Approach human	Within a 2 m radius, the horse’s head and neck move toward the client, reducing the distance between them for at least 3 s, with the possibility of the horse initiating physical contact.	Absent: 0 times throughout the session Occasionally: 1 to 2 times throughout the session Frequently: more than 2 times throughout the session	[36]
Snort	An audible exhalation through the nose lasting a few seconds, during which the mouth and nose muscles remain relaxed.	Absent: 0 times throughout the session Occasionally: 1 to 2 times throughout the session Frequently: more than 2 times throughout the session	[45,46,47,48]
Blow	A short, sudden, audible exhalation through the nose, accompanied by a closed mouth and tension in the muscles of the mouth and nose.	Absent: 0 times throughout the session Occasionally: 1 to 2 times throughout the session Frequently: more than 2 times throughout the session	[37,43]
Tight mouth	The mouth is tightly closed, with visible tension in the jaw muscles and the areas around the nose, lips, and corners of the mouth, sustained for at least 3 s.	Absent: 0% of the session Occasionally: 1 to 30% of the session Frequently: more than 30% of the session	[33,34,35,38,49]
Yawning	The mouth is held wide open for at least 3 s, often revealing the teeth, and occasionally the upper and lower jaws move from side to side.	Absent: 0 times throughout the session Occasionally: 1 to 2 times throughout the session Frequently: more than 2 times throughout the session	[23,36,39]
Licking and chewing	With relaxed mouth, nasal, and jaw muscles, the horse displays repetitive jaw movements mimicking food consumption, lasting at least 3 s and occasionally accompanied by visible tongue movements.	Absent: 0 times throughout the session Occasionally: 1 to 5 times throughout the session Frequently: more than 5 times throughout the session	[32,42]
Rolling	The horse moves on the ground, rolling from a sternal position to a lateral position, with all four legs lifting off the ground; rolling over the withers is not required.	Absent: 0 times throughout the session Occasionally: 1 to 2 times throughout the session Frequently: more than 2 times throughout the session	[39,43]
Defecate	The release of (in)digested feed through the anus.	Absent: 0 times throughout the session Occasionally: 1 to 2 times throughout the session Frequently: more than 2 times throughout the session	[50]
Self-grooming	Nibbling or manipulating its own skin with the mouth and teeth for at least 3 s.	Absent: 0 times throughout the session Occasionally: 1 to 2 times throughout the session Frequently: more than 2 times throughout the session	[39]
Pawing	A repetitive motion (at least twice) of one front leg, scraping the ground, another surface, or the air in a front-to-back movement.	Absent: 0 times throughout the session Occasionally: 1 to 2 times throughout the session Frequently: more than 2 times throughout the session	[39,43,50]
Snapping and biting	The horse uses its lips, mouth, or teeth to nibble or bite the client, another horse, or an object.	Absent: 0 times throughout the session Occasionally: 1 to 2 times throughout the session Frequently: more than 2 times throughout the session	[51]
Resting hindleg	One hind leg rests on the ground with the front of the hoof, while the horse’s weight is supported by the other three legs, causing the pelvis to dip on the side of the resting hind leg.	Absent: 0% of the session Occasionally: 1 to 30% of the session Frequently: more than 30% of the session	[52]

**Table 2:** Percentages of horses for different management factors.

	Equine-Assisted Coaching Horse (Percentage of Horses, *n* = 94)	Therapeutic Riding Horse (Percentage of Horses, *n* = 13)
**Housing**		
Individual stabled inside	27.0	75.0
Individual outside	1.4	0.0
Group housing inside	4.1	0.0
Group housing outside	50.0	25.0
Other	17.6	0.0
**Feeding management**		
*Ad libitum* roughage	58.8	53.8
Less than 6 consecutive hours without roughage per day	8.2	15.4
More than 6 consecutive hours without roughage per day	32.9	30.4
**Access to pasture/paddock**		
24 h a day	70.6	30.8
Between 6 and 24 h a day	23.5	38.5
Less than 6 h a day	5.9	30.8

**Table 3:** Percentage of sessions in which each behaviour was displayed frequently, occasionally or was absent during equine-assisted coaching sessions over a two-month period.

Behaviour Label	Frequently	Occasionally	Absent
Relaxed tail	65%	34%	2%
Ears forward	44%	50%	6%
Head low	42%	49%	9%
Approach human	42%	42%	16%
Ears active	38%	56%	6%
Licking and chewing	19%	66%	15%
Moving away	17%	47%	35%
Snort	15%	36%	49%
Resting hindleg	11%	45%	45%
Yawning	11%	24%	66%
Head high	10%	44%	45%
Self-grooming	7%	28%	64%
Tail swishing	5%	41%	54%
Pawing	5%	18%	77%
Snapping and biting	4%	14%	82%
Blow	3%	11%	85%
Tight mouth	3%	41%	57%
Defecate	3%	32%	65%
Rolling	2%	18%	80%
Ears flattened	1%	21%	77%
Head shaking	1%	43%	56%

**Table 4:** Percentage of sessions in which each behaviour was displayed frequently, occasionally or was absent during therapeutic riding sessions over a two-month period.

Behaviour Label	Frequently	Occasionally	Absent
Relaxed tail	87%	12%	1%
Ears active	54%	25%	21%
Ears forward	50%	41%	9%
Head low	25%	49%	25%
Head high	17%	24%	58%
Tight mouth	9%	32%	59%
Snort	9%	25%	66%
Snapping and biting	8%	18%	74%
Approach human	5%	31%	64%
Licking and chewing	5%	42%	53%
Ears flattened	4%	29%	67%
Tail swishing	4%	39%	57%
Defecate	3%	58%	39%
Head shaking	2%	38%	60%
Moving away	1%	13%	86%
Pawing	1%	5%	94%
Yawning	0%	5%	95%
Blow	0%	0%	100%
Rolling	0%	0%	100%
Resting hindleg	0%	1%	99%
Self-grooming	0%	3%	97%

**Table 5:** The upper and lower thresholds for 21 behaviours exhibited by horses during therapeutic riding and equine-assisted coaching sessions, derived from an evaluation exercise in which 28 experts assessed 11 fictional sessions for each behaviour. See for definition of the behaviour in Table 1.

Behaviour	Definition of Observed Frequently	Lower Threshold (Percentage of Sessions)	Upper Threshold (Percentage of Sessions)
Ears forward	More than 10% of a 45-min session	45%	87%
Ears flattened	More than 5 times in a 45-min session	43%	10%
Ears active	More than 10% of a 45-min session	37%	68%
Head high	More than 10% of a 45-min session	62%	16%
Head low	More than 10% of a 45-min session	40%	79%
Head shaking	More than 5 times in a 45-min session	53%	6%
Relaxed tail	More than 30% of a 45-min session	52%	83%
Tail swishing	More than 5 times in a 45-min session	56%	9%
Moving away	More than 2 times in a 45-min session	59%	26%
Approach human	More than 2 times in a 45-min session	32%	80%
Snort	More than 2 times in a 45-min session	16%	75%
Blow	More than 2 times in a 45-min session	56%	14%
Tight mouth	More than 30% of a 45-min session	36%	5%
Yawning	More than 2 times in a 45-min session	57%	13%
Licking and chewing	More than 5 times in a 45-min session	51%	20%
Rolling	More than 2 times in a 45-min session	50%	10%
Defecate	More than 2 times in a 45-min session	50%	10%
Self-Grooming	More than 2 times in a 45-min session	54%	19%
Pawing	More than 2 times in a 45-min session	67%	7%
Snapping and biting	More than 2 times in a 45-min session	55%	4%
Resting hindleg	More than 30% of a 45-min session	56%	15%

**Table 6:** Component loadings of Principal Component Analyses (PCA) performed on the presence of the frequently observed behaviours during 432 sessions of equine-assisted coaching with 85 horses. The components are labelled according to the behaviours that contributed significantly to each component. Notably, all behaviours showed positive contributions to their respective components.

Behaviour/Label	Comfortable	Tense	Release Tension	Restless	Agitated	Detached
	Component 1	Component 2	Component 3	Component 4	Component 5	Component 6
Ears forward	0.806					
Head low	0.737					
Ears active	0.709					
Relaxed tail	0.585					
Snort		0.788				
Blow		0.682	0.420			
Head shaking		0.645				
Rolling		0.535		0.549		
Tail swishing		0.477			0.479	
Defecate			0.790			
Yawning			0.630			
Licking and chewing			0.584			
Pawing			0.541	0.438		
Approach human				0.742		
Moving away				0.669		
Snapping and biting				0.591	0.423	
Ears flattened					0.797	
Tight mouth					0.690	
Resting hindleg						0.677
Self grooming						0.629
Variance explained	11.9%	11.5%	11.4%	10.4%	9.8%	8.0%
Cronbach’s α	0.60	0.55	0.56	0.52	0.44	0.29
Eigenvalue	3.569	2.700	1.860	1.738	1.523	1.202

**Table 7:** Component loadings of Principal Component Analyses (PCA) performed on the presence of the frequently observed behaviours during 398 therapeutic horse-riding sessions with 13 horses. The components are labelled according to the behaviours that contributed significantly to each component. Notably, all behaviours showed positive contributions to their respective components.

Behaviour/Label	Building Tension	Agitated	Release Tension	Comfortable
	Component 1	Component 2	Component 3	Component 4
Resting hindleg	0.905			
Moving away	0.781			
Tight mouth	0.653	0.519		
Head high	0.528			
Licking and chewing	0.404		0.704	
Snapping and biting		0.902		
Ears flattened		0.843		
Tail swishing		0.571		
Blow			0.895	
Ears forward				0.851
Ears active				0.814
Variance explained	22.0%	21.2%	14.8%	14.2%
Cronbach’s α	0.602	0.652	0.536	0.485
Eigen value	3.538	1.904	1.391	1.121

## Data Availability

Data are available on request.
